# Timing flexibility of oral NEPA, netupitant-palonosetron combination, administration for the prevention of chemotherapy-induced nausea and vomiting (CINV)

**DOI:** 10.1007/s00520-019-4640-8

**Published:** 2019-01-26

**Authors:** Sally Baron-Hay, Matti Aapro, Alberto Bernareggi, Lee Schwartzberg

**Affiliations:** 1 Royal North Shore Hospital, St Leonards, NSW Australia; 2 Genolier Cancer Centre, Clinique de Genolier, Genolier, Switzerland; 3 Helsinn Healthcare SA, Lugano, Switzerland; 4 West Cancer Center, Memphis, TN USA

**Keywords:** Chemotherapy-induced nausea and vomiting, CINV, NEPA, Netupitant, Palonosetron, Administration timing

## Abstract

**Purpose:**

The administration timing of antiemetic and chemotherapeutic regimens is often determined by regulatory indications, based on registration studies. Oral NEPA, fixed combination of the neurokinin-1 receptor antagonist (NK_1_RA) netupitant and the 5-hydroxytryptamine-3 RA (5-HT_3_RA) palonosetron, is recommended to be administered approximately 60 min before chemotherapy. Reducing chair time for chemotherapy administration at oncology day therapy units would improve facility efficiency without compromising patient symptom management. The objective was to determine if oral NEPA can be administered closer to chemotherapy initiation without compromising patient symptom management.

**Methods:**

NK_1_ receptor occupancy (NK_1_RO) time course in the brain was determined using positron emission tomography; netupitant and palonosetron plasma concentration-time profiles were described by pharmacokinetic (PK) models; and the rate, extent, and duration of RO by netupitant and palonosetron were predicted by pharmacodynamic modeling. Clinical efficacy data from a pivotal study in cisplatin and oral NEPA-receiving patients were reviewed in the context of symptom management.

**Results:**

Striatal 90% NK_1_RO, assumed to correlate with NK_1_RA antiemetic efficacy, was predicted at netupitant plasma concentration of 225 ng/mL, reached at 2.23 h following NEPA administration. Palonosetron 90% 5-HT_3_RO was predicted at a 188-ng/L plasma concentration, reached at 1.05 h postdose. The mean time to first treatment failure for the 1.5% of NEPA-treated patients without complete response receiving highly emetogenic chemotherapy was 8 h. Antiemetic efficacy was sustained over 5 days despite the expected decrease of NK_1_RO and 5-HT_3_RO.

**Conclusions:**

Results suggest that administering oral NEPA closer to initiation of cisplatin administration would provide similar antiemetic efficacy. Prospective clinical validation is required.

## Introduction

Chemotherapy-induced nausea and vomiting (CINV) remains one of the most distressing side effects of emetogenic chemotherapy and can negatively impact quality of life and overall survival of cancer patients [1–3]. Advances in antiemetic research have improved the therapeutic options for the prevention of CINV [4]. However, complete control of emesis, and especially of nausea, is still not achieved in many cancer patients [5, 6]. The American Society of Clinical Oncology guidelines [7], National Comprehensive Cancer Network [8], and the Multinational Association of Supportive Care in Cancer/European Society for Medical Oncology guidelines [9] recommend the triple combination of a 5-hydroxytryptamine-3 receptor antagonist (5-HT_3_RA), a neurokinin-1 (NK_1_)RA, and dexamethasone for CINV prevention associated with highly emetogenic chemotherapy (HEC), anthracycline-cyclophosphamide (AC)-based regimens, and carboplatin regimens, with the addition of olanzapine to the triple combination discussed under specific conditions. Finally, patients treated with moderately emetogenic chemotherapy should receive a 5-HT_3_RA and dexamethasone [7–9], or the triplet NK_1_RA–5-HT_3_RA–dexamethasone combination if they present with additional risk factors or for whom 5-HT_3_RA and dexamethasone alone fail [8].

CINV is classified as acute or delayed, depending on the timing of its occurrence after the start of chemotherapy administration [10]. The acute phase is defined as the 24 h following chemotherapy and is largely mediated by serotonin activation of 5-HT_3_ receptors in the intestine, and, to a lesser extent, by activation of centrally located 5-HT_3_ receptors in the area postrema and nucleus tractus solitarius [11, 12], while the delayed phase is defined as the 25–120 h after chemotherapy and is predominantly driven by substance P activation of NK_1_ receptors in the area postrema and the nucleus tractus solitarius [10]. Crosstalk between 5-HT_3_ and NK_1_ receptors could also contribute to CINV [13]. Generally, 5-HT_3_RAs have proven highly effective in controlling CINV in the acute phase but poor at control in the delayed phase [14, 15]. Conversely, NK_1_RAs are most effective in the prevention of CINV during the delayed phase [4]. Several chemotherapeutic agents, such as cisplatin, can induce both acute and delayed CINV [16].

Antiemetic prophylaxis is administered prior to the start of chemotherapy. Among the factors affecting the administration convenience of the chosen antiemetic regimen are the precise timing of administration, the number of agents, the number of doses, and the number of days of treatment. Minimizing the time lapse between the administration of antiemetic regimens and of chemotherapeutic treatments could benefit health care centers and patients. With chair time being a significant issue for busy oncology day therapy units, reducing the time patients occupy a treatment chair for chemotherapy administration could improve facility efficiency without compromising patient symptom management.

Oral NEPA is the first fixed-combination antiemetic, composed of the highly selective NK_1_RA netupitant (300 mg) and the pharmacologically and clinically distinct 5-HT_3_RA palonosetron (0.5 mg) [4, 13]. Oral NEPA uniquely targets two critical emetic pathways and provides protection against both acute and delayed CINV [17–19]. In the registration trials, oral NEPA plus dexamethasone offered superior CINV control in patients receiving cisplatin- and AC-based chemotherapy, compared with oral palonosetron plus dexamethasone [17, 18]. In these studies, NEPA and palonosetron were both administered as a single oral dose approximately 60 min prior to chemotherapy on day 1. The timing for NEPA administration was chosen on the basis of the design used in prior registration studies of aprepitant, the first approved NK_1_RA [20, 21], while no clinical data supporting this choice are available. As an alternative for patients who cannot swallow oral medication, an intravenous formulation of NEPA (fosnetupitant 235 mg/palonosetron 0.25 mg) administered as a 30-min infusion before chemotherapy has been developed and approved by the US Food and Drug Administration [22] for patients receiving HEC, and it is currently being evaluated in the AC setting.

The convenience of NEPA dosing could be improved by allowing flexibility in the timing of its administration. The start of antiemetic activity is assumed to be related to the time elapsed from drug administration to occupancy of target receptors above a therapeutic threshold, in the relevant regions of the central (CNS) and peripheral (PNS) nervous system. A positron emission tomography (PET) study in humans using aprepitant found that the highest concentration of NK_1_ receptors in the brain was in the striatum and demonstrated a good correlation between > 90% NK_1_ receptor occupancy (RO) in the striatum at therapeutic doses and antiemetic efficacy [23]. Consequently, 90% RO in the striatum has become a recognized threshold correlating with NK_1_RA efficacy [23] and is an accepted surrogate marker for effective NK_1_RA interaction with NK_1_ receptors in the area postrema and nucleus tractus solitarius. In the present analysis, the same > 90% 5-HT_3_RO in relevant tissues of the CNS and PNS [12, 24] was assumed as the threshold required for palonosetron antiemetic effect.

Data from previous pharmacokinetic (PK) and pharmacodynamic (PD) studies carried out during the development of NEPA were used to establish here a PK/PD model-based analysis of NK_1_- and 5-HT_3_RO in their respective relevant tissues. Clinical data from a pivotal trial in patients receiving cisplatin-based chemotherapy [17] were evaluated to establish if a correlation could be made between the PK/PD model estimates and the clinical data. As cisplatin is ranked among the most emetogenic chemotherapeutic agents and with emetic activity in the acute and delayed periods [16, 25, 26], this would provide data applicable to broader chemotherapeutic regimens.

## Methods

### Study design (Fig. 1) [17, 27, 28]

Data used for PK/PD modeling of netupitant and palonosetron in this analysis were obtained from previous preclinical and clinical studies performed during the development of oral NEPA and palonosetron.

**Fig. 1:**
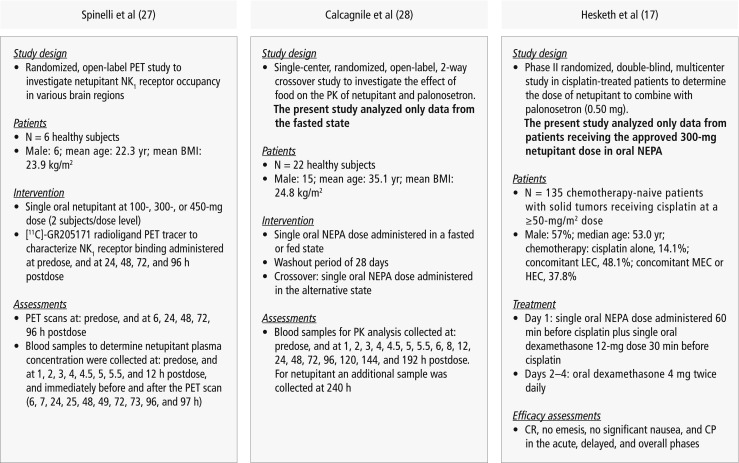
Design of studies included for the PK/PD modeling and for the correlation with antiemetic clinical efficacy [17, 27, 28]. BMI, body mass index; CP, complete protection (CR and no significant nausea); CR, complete response (no emesis, no rescue medication); HEC, highly emetogenic chemotherapy; LEC, low emetogenic chemotherapy; MEC, moderately emetogenic chemotherapy; NK_1_, neurokinin-1; oral NEPA, 300 mg netupitant/0.50 mg palonosetron; PD, pharmacodynamic; PET, positron emission tomography; PK, pharmacokinetics

PD data characterizing the interaction of netupitant with NK_1_ receptors in the brain were from a single-dose, open-label PET study in six healthy adult males randomized to receive oral netupitant at 100-, 300-, or 450-mg dose (two subjects/dose) [27]. Together with oral netupitant, subjects received a highly selective, high-affinity NK_1_RA PET tracer, [^11^C]-GR205171, as an intravenous bolus injection at baseline, and at approximately 6, 24, 48, 72, and 96 h after dosing with netupitant. The injections were followed by 60-min PET scans. This procedure allowed the evaluation of netupitant brain penetration, the rate and extent of netupitant interaction with NK_1_ receptors in different brain regions, and the receptor washout rate. From this PET study, a maximum effect (*E*_max_) model was established to relate NK_1_RO as a function of netupitant plasma concentration. Through the model, the time required to achieve the 90% NK_1_RO in the striatum was predicted.

PD data characterizing the interaction of palonosetron with 5-HT_3_ receptors in tissues were from preclinical studies in NG-108-15 [29] and in HEK 293 cell membranes stably expressing 5-HT3_A_ and 5-HT3_B_ receptors [30].

PK modeling of netupitant and palonosetron plasma concentration-time profiles was based on two-compartment model fitting to mean curves observed in an open-label, randomized phase I study in 22 healthy adults aimed at testing the effect of food on the PK of netupitant and palonosetron [28]. The subjects received single doses of oral NEPA in a fed or fasted state in the initial treatment period and in the alternative state in the following treatment period after a washout of 28 days. Mean netupitant and palonosetron plasma concentration-time curves used for PK modeling were from subjects receiving oral NEPA in the fasted state.

Netupitant and palonosetron PK/PD modeling results were correlated with clinical data from the multinational, randomized, double-blind, parallel group, phase II study in 694 chemotherapy-naive cancer patients scheduled to receive cisplatin-based HEC [17]. This study compared antiemetic efficacy and safety of three different oral doses of netupitant (100, 200, and 300 mg) plus 0.5 mg palonosetron, all given on day 1. A standard 3-day aprepitant plus intravenous ondansetron 32-mg regimen was included as an exploratory arm. All patients received a single oral dose of 12 mg dexamethasone 30 min before cisplatin on day 1 and 4 mg twice daily on days 2–4.

The primary efficacy endpoint was complete response (CR; no emesis, no rescue medication) during the overall phase (0–120 h following chemotherapy). Efficacy analysis results from 135 chemotherapy-naive patients receiving 300 mg netupitant plus 0.5 mg palonosetron (the approved oral NEPA dose) 60 min before cisplatin on day 1 were used to establish clinical correlations with the outcomes from PK/PD modeling analyses.

Detailed design, methods, and patient eligibility criteria for the clinical studies have been published previously [17, 27, 28]. For each, the relevant study protocols were approved by the corresponding ethical review committees, and sites participating in the studies followed the International Conference on Harmonization E6 Good Clinical Practice guidelines, Declaration of Helsinki principles, and local laws and regulations.

### Netupitant PK/PD analysis

In the PET study, the extent of NK_1_RO in different brain regions (striatum, lateral and medial temporal cortex, occipital and frontal cortex, and anterior cingulate) was determined by PET scans following single oral dose administration of netupitant [27].

Blood samples for the determination of netupitant plasma concentrations were collected at the following time points: predose 1, 2, 3, 4, 4.5, 5, 5.5, and 12 h postdose, immediately before the PET scan (6, 24, 48, 72, and 96 h postdose), and immediately after the PET scan (7, 25, 49, 73, and 97 h postdose). Parameter values and the precision of the estimates are reported in the paper by Spinelli et al. [27].

For all subjects, individual NK_1_RO observations in the striatum and other brain regions were correlated with the respective netupitant plasma concentrations by sigmoid *E*_max_ modeling (Eq. 1):

1 $$ \mathrm{RO}\ \left(\%\right)=\frac{E_{max}\times {C}^{\upgamma}}{EC_{50}^{\upgamma}+{C}^{\upgamma}} $$

where *E*_max_ is the maximal NK_1_RO, EC_50_ is the plasma concentration at which 50% of *E*_max_ is reached, *C* is the netupitant plasma concentration at any time, and *γ* is a slope parameter reflecting the shape of the curve. The values of *E*_max_, EC_50_, and *γ* for each brain region were estimated by fitting the sigmoid *E*_max_ model to the experimental RO values as a function of netupitant plasma concentrations for all subjects and all doses simultaneously, using the software WinNonlin Professional Edition Version 4.1.b (Pharsight Corporation, Mountain View, CA).

The netupitant plasma concentration required to achieve 90% NK_1_RO, *C*_90%_, in the striatum was then predicted by Eq. 2, derived from Eq. 1:

2 $$ {C}_{90\%}=\sqrt[\gamma ]{\frac{90\%\times {EC}_{50}^{\gamma }}{E_{max}-90\%}} $$

The time required to reach *C*_90%_ after administration of 300-mg netupitant was estimated through the PK model (Eq. 3) obtained by fitting a two-compartment open model, with first-order absorption, first-order elimination and lag time, to mean plasma netupitant concentration using the PK software Phoenix WinNonlin version 6.4 (Certara, Princeton, NJ).

3 $$ {C}_t=A\times {e}^{-{\uplambda}_1\times t}+B\times {e}^{-{\uplambda}_2\times t}-C\times {e}^{-{K}_{01}\times t} $$

where *C*_*t*_ represents netupitant plasma concentration at any time, *A*, *B*, and *C* are hybrid constants, *λ*_1_ and *λ*_2_ are disposition rate constants, *K*_01_ is the absorption rate constant, and *t* is time. Mean plasma netupitant concentration-time curves were obtained from 22 healthy adults enrolled in the above-mentioned PK study [31]. The two-compartment model described by Eq. 3 was fitted to mean plasma concentration-time data of subjects in fasted state by iterative nonlinear regression analysis based on the minimization of the objective function until convergence. The weighted least squares analysis was performed using 1/*C*_(pred)_^2^ as a weighting factor, where *C*_(pred)_ is the predicted concentration. Goodness of fit was established on the basis of plots of observed vs. predicted concentrations, plots of weighted residuals, and conventional criteria, including the weighted sum of squared residuals, the Akaike Information Criterion, and the Schwarz Bayesian Criterion.

### Palonosetron PK/PD analysis

Palonosetron is a potent 5-HT_3_RA that exhibits allosteric binding and positive cooperativity upon binding to 5-HT_3_ receptors in HEK 293 cells [30]. In saturation-binding studies in NG-108-15 cell membranes, palonosetron showed a mean affinity (pK_i_) value of 10.45 M at the 5-HT_3_ receptor [29]. Assuming competitive inhibition, the palonosetron EC_50_ can be assumed to be approximately twofold the *K*_*i*_ [32]. Hence,

$$ {\mathrm{EC}}_{50}=2\times {10}^{-10.45}\mathrm{M}=\kern0.5em 0.071\ \mathrm{nM}=21\ \mathrm{ng}/\mathrm{L} $$

Interaction kinetics was modeled using Eq. 1, where *E*_max_ is the maximum palonosetron 5-HT_3_RO, assumed to be 100%, EC_50_ is the palonosetron plasma concentration at which 50% *E*_max_ is achieved, *C* is palonosetron concentration in plasma at any time, and *γ* is a slope parameter, assumed to be 1 (the sigmoid *E*_max_ model reduces to a simple *E*_max_ model).

The percentage of 5-HT_3_RO was simulated as a function of palonosetron concentration after oral administration of 0.5-mg palonosetron. Mean palonosetron plasma concentration-time data from 22 healthy adults enrolled in the Calcagnile et al. [28] study, who received a single oral NEPA dose in the fasted state, were applied to Eq. 3, where *C*_t_ represents palonosetron plasma concentration at any time.

### Pivotal phase II clinical study [17]

For the collection of efficacy data, patients completed a diary through the first 120 h after receiving cisplatin, including the following information: timing and duration of each emetic episode, severity of nausea using a 100-mm horizontal visual analog scale, and use of concomitant and rescue medication. In this analysis, the percentages of patients with CR, without emesis, and with “no significant nausea” (NSN) were calculated for the acute period and for each day after (days 2–5), for the full analysis set. The mean time to first emetic episode and the time to treatment failure (time to the first emetic episode or use of rescue medication, whichever occurred first) were determined using the patient-reported data.

## Results

### Netupitant PK/PD modeling

The sigmoid *E*_max_ model parameters from the PET study [27] indicated *E*_max_ values greater than 90% in most of investigated brain regions. Estimates of EC_50_ and *γ* ranged from 0.2 to 10.2 μg/L and from 0.5 to 1.2 μg/L, respectively, and were characterized by good precision in the striatum. In other brain areas, the limited number of experimental points in the ascending part of the RO vs. plasma concentration curves affected the precision of the EC_50_ and *γ* estimates.

PK model parameters reported in Table 1 [28] were estimated by fitting a two-compartment open model (Eq. 3) to the mean plasma concentration-time curves of netupitant from healthy adults receiving 300-mg netupitant as oral NEPA fixed combination [28] and were used to simulate the netupitant plasma concentration-time profile at any time following administration of oral NEPA.

**Table 1:** PK parameters for netupitant and palonosetron estimated from two-compartmental modeling of plasma concentration-time data

	Netupitant			Palonosetron		
Parameter	Units	Estimate	CV%	Units	Estimate	CV%
*A*	μg/L	521.1	10.4	ng/L	536.3	21.9
*B*	μg/L	86.9	12.6	ng/L	335.6	42.9
*K*_01_	h^−1^	0.95622	32.0	h^−1^	0.70045	13.1
*λ*_1_	h^−1^	0.07144	13.9	h^−1^	0.04955	32.8
*λ*_2_	h^−1^	0.00673	11.6	h^−1^	0.01294	26.8
*t*_lag_	h	1.62	7.4	h	0.67	5.9

Mean plasma concentration-time curves of netupitant and palonosetron from 22 healthy adults who received oral NEPA in the fasted state were used for modeling [28]

*λ*_1_, *λ*_2_, disposition rate constants; *A*, *B*, hybrid coefficients; CV, coefficient of variation; *K*_01_, absorption rate constant; PK, pharmacokinetics; *t*_lag_, lag time

**Fig. 2:**
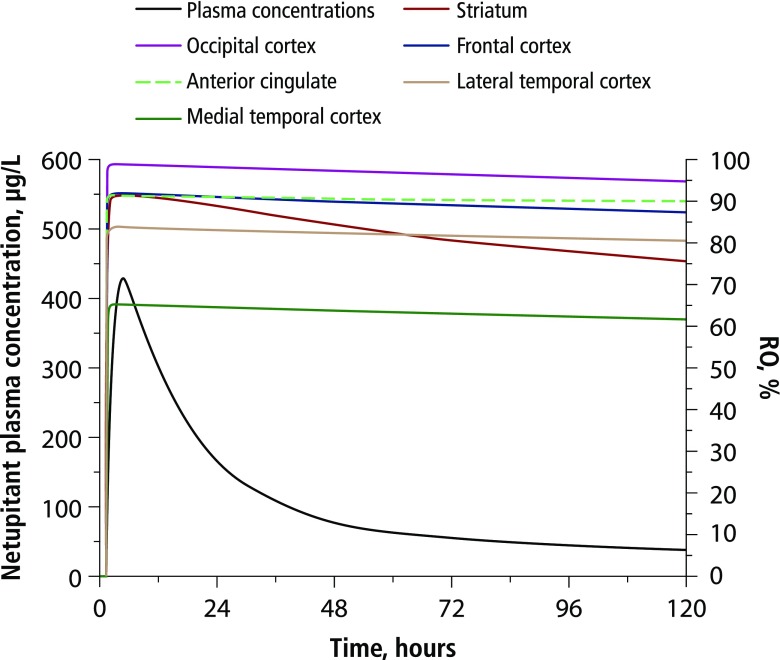
Model-predicted netupitant NK_1_RO in different brain regions and netupitant plasma concentrations as a function of time after administration of 300-mg netupitant. NK_1_RO, neurokinin-1 receptor occupancy

The PK/PD correlation between predicted netupitant NK_1_RO in all tested brain regions and predicted netupitant plasma concentrations following 300-mg oral netupitant is presented in Fig. 2. Higher and longer-lasting NK_1_RO were predicted in the occipital cortex, the anterior cingulate, and the frontal cortex, where netupitant RO was greater than or close to 90% up to 120 h postdosing. In the striatum, netupitant NK_1_RO was predicted to exceed 90% up to approximately 24 h after drug administration, then to decline slowly, reaching 75–80% RO on day 5 postdosing. Netupitant washout from the blood compartment was predicted to be faster than from all brain regions, confirming the high affinity of netupitant for NK_1_ receptors in the brain.

PD model-predicted NK_1_RO (Fig. 2) is consistent with experimental values determined by PET after oral administration of 300-mg netupitant [27]. Using the netupitant PK model parameters reported in Table 1 [28], the 90% NK_1_RO in the striatum was predicted to be attained at a netupitant plasma concentration of 225 ng/mL, reached at 2.23 h after administration of oral NEPA, i.e., earlier than the netupitant peak time, estimated at ∼ 6 h. In addition, 90% NK_1_RO would be reached within 3 h in other brain regions such as the occipital cortex, the frontal cortex, and the anterior cingulate (Fig. 2).

### Palonosetron PK/PD modeling

PK model parameters reported in Table 1 [28] were estimated by fitting a two-compartment open model (Eq. 3) to the mean plasma concentration-time curves of palonosetron from healthy adults receiving 0.5-mg palonosetron as oral NEPA fixed combination [28]. These parameters were used to simulate the palonosetron plasma concentration-time profile at any time following administration of oral NEPA.

PD model-predicted palonosetron 5-HT_3_RO as a function of PK model-predicted palonosetron plasma concentrations after administration of 0.5-mg palonosetron as oral NEPA indicated that 90% 5-HT_3_RO in tissues is expected to be attained at a palonosetron plasma concentration of 188 ng/L, reached at 1.05 h after administration of oral NEPA, i.e., earlier than the palonosetron peak concentration of 693 ng/L, estimated at 5.2 h.

**Fig. 3:**
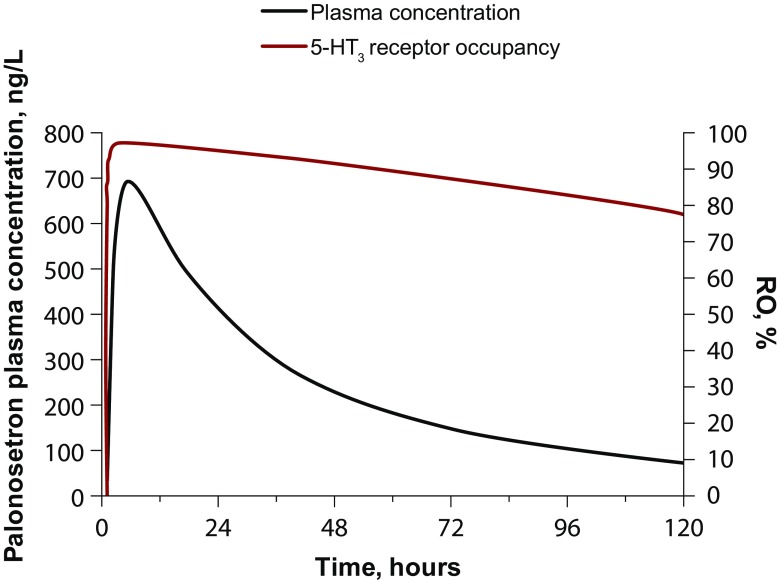
Model-predicted palonosetron 5-HT_3_RO and predicted palonosetron plasma concentrations as a function of time after administration of 0.5-mg palonosetron. 5-HT_3_RO, 5-hydroxytryptamine-3 receptor occupancy

The PK/PD correlation between predicted palonosetron 5-HT_3_RO and predicted palonosetron plasma concentrations as a function of time is presented in Fig. 3. Palonosetron 5-HT_3_RO was predicted to exceed 90% up to approximately 3 days after drug administration, and then it declined slowly, reaching a RO of approximately 80% on day 5 postdosing. Palonosetron washout from the blood compartment was predicted to be faster than from 5-HT_3_ receptors in tissues because of the high affinity of palonosetron for 5-HT_3_ receptors.

### Pivotal phase II clinical study

The time to first treatment failure for any patient treated with oral NEPA was 8 h, with a mean time to treatment failure of 114.2 h [17]. The time to the first emetic episode for any oral NEPA-treated patient was also 8 h, with a mean time to first emesis of 114.4 h. The time to the first administration of rescue medication was 95 h, and the mean time was 119.8 h. In the acute phase, for patients receiving NEPA prophylaxis, the rates of no emesis, NSN, and CR were 99% for each, with daily rates of no emesis and NSN of ≥ 95% on days 2–5.

## Discussion

The prevention of acute CINV is mainly dependent on inhibition of 5-HT_3_ receptors by 5-HT_3_RAs, while delayed CINV control is associated with NK_1_ receptor inhibition by NK_1_RAs [4]. The pivotal clinical study included in this analysis [17] previously demonstrated the superiority of oral NEPA vs oral palonosetron in the rate of acute CR, suggesting that the NK_1_RA component of the fixed combination, netupitant, may also contribute to the prevention of CINV in the acute period. The present analysis predicted that palonosetron may occupy 90% of 5-HT_3_ receptors at a plasma concentration of 188 ng/L within 1.05 h after dosing, while netupitant may reach the therapeutic threshold of 90% RO in the striatum [23] at a plasma concentration of 225 ng/mL which is reached as early as 2.23 h after administration. These results further support the role of both components of NEPA in CINV control during the acute phase.

Cisplatin-associated acute nausea and vomiting has been shown to start within the first 4 h after initiation of chemotherapy, and to reach a peak between 4 to 10 h [31]. Here, > 90% of 5-HT_3_ and of striatal NK_1_ receptors were predicted to be occupied at 1.05 and 2.23 h, respectively, postadministration of oral NEPA, thus before the start of emetic episodes associated with cisplatin treatment. Accordingly, clinical data showed that the time to first treatment failure following cisplatin administration for any patient among the 135 patients in the oral NEPA group was 8 h. Therefore, reducing the time of administration of NEPA to less than 1 h prior to the administration of cisplatin would not be expected to impact its antiemetic efficacy in the acute phase. In addition, > 90% occupancy of 5-HT_3_ and of striatal NK_1_ receptors was predicted to be sustained over approximately 72 and 24 h, respectively, after oral NEPA administration. This prolonged RO also suggests that increasing the time of administration of NEPA to more than 1 h before cisplatin administration would not affect its antiemetic activity.

The antiemetic activity of oral NEPA is sustained in the delayed phase, with a daily CR rate ranging from 96 to 98% on days 2–5 [17]. Although a 90% occupancy of striatal NK_1_ receptors has been assumed as the threshold to reach antiemetic activity [23], it seems that this level does not need to be sustained over the entire delayed period to exert antiemetic control, since on day 4 a 98% CR rate was attained [17] with an estimated NK_1_RO in the striatum of 78% (Fig. 2). Noteworthy, in other brain regions such as the occipital cortex and the anterior cingulate, 90% NK_1_RO was exceeded up to 120 h after NEPA administration. Previous studies have shown that palonosetron and netupitant can act synergistically on the inhibition of the substance P signaling pathway [13, 33]. Palonosetron can inhibit crosstalk between the NK_1_ and 5-HT_3_ receptor signaling pathways and induce 5-HT_3_ receptor internalization, which may result in prolonged inhibition of NK_1_ and 5-HT_3_ receptor function/signaling pathways [13].

Overall, the results presented here suggest a potential for flexibility in the administration timing of NEPA administered immediately before chemotherapy. Administration of NEPA closer to the time of chemotherapy would most likely not affect delayed CINV control, as maintaining ≥ 90% NK_1_RO in the striatum, surrogate marker for effective NK_1_RA interaction in the area postrema and the nucleus tractus solitarius, does not seem to be required for antiemetic efficacy.

Some limitations of this study include the small number of subjects involved in the PET study with netupitant; the fact that the PET study analyzed the interaction with NK_1_ receptors following administration of netupitant as single agent; the assumption of the adequacy of a sigmoid *E*_max_ model to describe the interaction of palonosetron with the 5-HT_3_ receptor; and the assumption of the 90% 5-HT_3_RO threshold to establish 5-HT_3_RA antiemetic activity for palonosetron. In addition, the data used to develop the PK and PD models, as well as the clinical trial results used to establish potential correlations with clinical antiemetic efficacy, were obtained from independent studies analyzing different subject or patient populations. These limitations and assumptions appear to be acceptable in light of the good correlation between model-predicted (Fig. 2) and observed NK_1_RO [27] in the different brain regions. In addition, the degree of NK_1_ and 5-HT_3_RO correlated well with the described antiemetic effects of NEPA in clinical trials. This retrospective analysis using PK/PD modeling allows generation of accurate predictions about the clinical effects of the timing of oral NEPA administration rapidly and in a noncostly manner that can be used as guidance for optimization of antiemetic administration in future clinical studies. Ultimately, a prospective clinical validation of these results would be required. In fact, a noninferiority study (in terms of CR rate) in cancer patients to examine two different administration times of NEPA relative to the first dose of HEC has been approved and will shortly begin accrual.

In conclusion, the PK/PD modeling and clinical data presented herein suggest that moving the timing of oral NEPA administration closer to chemotherapy initiation would probably not result in a loss of efficacy and could enhance the convenience of the administration. Prospective clinical validation is warranted to confirm these indications.
